# Estimation of Japanese encephalitis virus infection prevalence in mosquitoes and bats through nationwide sentinel surveillance in Indonesia

**DOI:** 10.1371/journal.pone.0275647

**Published:** 2022-10-12

**Authors:** Ajib Diptyanusa, Elisabeth Siti Herini, Soedarmanto Indarjulianto, Tri Baskoro Tunggul Satoto

**Affiliations:** 1 Doctoral Study Program of Health and Medical Sciences, Faculty of Medicine, Public Health and Nursing, Universitas Gadjah Mada, Yogyakarta, Indonesia; 2 World Health Organization Indonesia Country Office, Jakarta, Indonesia; 3 Department of Child Health, Faculty of Medicine, Public Health and Nursing, Universitas Gadjah Mada, Yogyakarta, Indonesia; 4 Department of Internal Medicine, Faculty of Veterinary Medicine, Universitas Gadjah Mada, Yogyakarta, Indonesia; 5 Department of Parasitology, Faculty of Medicine, Public Health and Nursing, Universitas Gadjah Mada, Yogyakarta, Indonesia; University of Oklahoma Norman Campus: The University of Oklahoma, UNITED STATES

## Abstract

Indonesia belongs to endemic areas of Japanese encephalitis (JE), yet data regarding the true risk of disease transmission are lacking. While many seroprevalence studies reported its classic enzootic transmission, data related to the role of bats in the transmission of JE virus are limited. This current study aimed to identify the potential role of bats in the local transmission of the JE virus to aid the ongoing active case surveillance in Indonesia, in order to estimate the transmission risk. Mosquitoes and bats were collected from 11 provinces in Indonesia. The detection of the JE virus used polymerase chain reaction (PCR). Maps were generated to analyze the JE virus distribution pattern. Logistic regression analysis was done to identify risk factors of JE virus transmission. JE virus was detected in 1.4% (7/483) of mosquito pools and in 2.0% (68/3,322) of bat samples. Mosquito species positive for JE virus were *Culex tritaeniorhynchus* and *Cx*. *vishnui*, whereas JE-positive bats belonged to the genera *Cynopterus*, *Eonycteris*, *Hipposideros*, *Kerivoula*, *Macroglossus*, *Pipistrellus*, *Rousettus*, *Scotophilus* and *Thoopterus*. JE-positive mosquitoes were collected at the same sites as the JE-positive bats. Collection site nearby human dwellings (AOR: 2.02; *P =* 0.009) and relative humidity of >80% (AOR: 2.40; *P =* 0.001) were identified as independent risk factors for JE virus transmission. The findings of the current study highlighted the likely ongoing risk of JE virus transmission in many provinces in Indonesia, and its potential implications on human health.

## Introduction

Mosquito-borne diseases belong to the group of most common infectious diseases in tropical and subtropical countries [1, 2]. Among these diseases, Japanese encephalitis (JE) has been reported to be the major cause of viral encephalitis in many countries in Asia [3]. Traditionally, the enzootic cycle of JE virus transmission involves mainly *Culex* mosquitoes as the vectors and pigs as the reservoir hosts [4], while humans serve as dead-end hosts [5]. Indonesia belongs to the endemic areas of JE, with sporadic cases reported annually since the year 1960 in various provinces [6–9]. The sentinel surveillance of human cases of JE in 11 provinces in Indonesia conducted in 2016 revealed a total of 37 seropositive JE cases from 243 cases of acute encephalitis syndrome, along with 5 lethal cases [10]. Remarkably, some of these clinical cases were reported in areas without the presence of pig farming, hence the presence of other potential reservoir hosts playing a role in the transmission of JE virus in these areas remains debatable [11]. In the past few decades, the role of bats in arbovirus transmission has been elucidated in many studies [12, 13], including in the detection of antibodies against arboviruses such as dengue, chikungunya, and JE [14–16].

The risk of JE virus transmission intensifies with the shifts in land use, especially areas suitable for habitats of mosquitoes [17]. Additionally, alterations in ambient temperature, relative humidity and wind velocity may play a role in the transmission of JE virus [18, 19], since these factors mostly affect the natural habitat, distribution, and behavior of vector mosquitoes [20]. Measurement of climatic factors along with mapping of high-risk areas of disease transmission are in line with the epidemiological surveillance, and these activities play a major role in mosquito-borne disease control and prevention [21, 22].

The One Health concept emphasizes the dense relationships among the health of humans, animal and environment, and explains that multidisciplinary approaches in managing JE should seriously be encouraged [23, 24]. The current study aimed to apprise the distribution of JE virus in mosquitoes and bats as parts of epidemiological surveillance of JE in Indonesia. The viremia profiling in bats may be used to estimate the potential role of bats as the reservoir hosts for JE virus. This study depicts the national surveillance of JE virus in mosquitoes and bats in Indonesia, of which some data from West Kalimantan province have been mentioned elsewhere [25]. The implementation of the One Health concept in regard to environmental health is assessed through stratification of the ecosystem via sample collection and the measurement of climatic factors. The results of the current study will be beneficial in providing vast additional data that can aid the formulation of policies concerning JE control and prevention, particularly vector and reservoir control and habitat conservation, JE vaccination campaign, and the expansion of the ongoing JE surveillance program.

## Methods

This descriptive cross-sectional study was conducted in collaboration with the Ministry of Health of Republic of Indonesia through its nationwide disease vector and reservoir surveillance program of Indonesia called *Rikhus Vektora* that was conducted from year 2015 through 2018. A total of 11 provinces were selected as study locations, and were determined based on the previous reports of human clinical cases of JE by the local health offices and previous studies [6, 26, 27], including West Sumatra, Riau, West Java, Yogyakarta, East Java, West Kalimantan, Bali, North Sulawesi, West Nusa Tenggara, East Nusa Tenggara, and Papua. The results of our pilot study conducted in West Kalimantan province have been published elsewhere [25], however, few data will be mentioned in this study in order to give the whole picture of JE virus infection in mosquitoes and bats in Indonesia. Sampling of bats and mosquitoes was purposively performed in 3 districts in each corresponding province and represents 3 different ecosystems: forest, coastal, and urban areas. Collection sites also include locations nearby and far from human dwellings, such as forest fringe and middle of forest and rural and urban areas. The different ecosystems of sample collection may facilitate the presence of species diversity, hence reflecting different transmission risk. The determination of sample collection sites was also done according to previous reports of human JE cases in national surveillance [10, 26]. Total sample collection was performed for 12 hours from 6 p.m. to 6 a.m. for 3 consecutive days in every study location. We recorded the coordinates of sample collection sites, along with the hourly temperature, relative humidity, and wind velocity.

### Operational definitions

We defined forests as homogenous or heterogenous plantation areas of either primary or secondary origin. Coastal area was defined as either beach, marshes, or tidal areas, while urban area was described as a residential environment that consists of more than one housing unit. Location in proximity with humans was defined as the presence human dwelling complex within 3 km of collection sites.

### Mosquito collection

Mosquitoes were collected using either human landing collection or animal-baited traps [28, 29]. We determined the mosquito species using morphological identification key [30]. We pooled about 1 to 25 mosquitoes into a single pool according to species, collection method, and time of collection. To preserve viral RNA, we put 500 μl of RNA later reagent (RNAlater, Thermo Fisher, USA) into each of the pooled mosquito tubes and the tubes were kept in 4°C prior to RNA extraction.

### Bat collection

Mist net and harp net were used to capture bats in the study sites [31, 32]. From the captured samples, we first identified any pregnant or lactating bats with dependent young to be released and excluded from the study. Prior to species identification and blood collection we performed anesthesia to the captured bats. Inhaled anesthesia was done using weight-dependent dose of isofluran. Bat sample was individually kept inside a recovery box after anesthesia, within which food and water was given. After the bats were fully recovered, they were released where they were previously captured. None of the captured bat sample was sacrificed. An identification key for Asian bats was used to identify bat species [33, 34]. Blood samples collected from the brachial vein of the bats were put onto 125 μl FTA card (Whatman, Merck, Germany) and kept in room temperature (20°C to 25°C) prior to molecular detection of JE virus.

### Molecular detection of JE virus

Collected mosquitoes in RNA later solution was dissected into head-and-thorax preparations and were put into different microtubes. A total of 500 μl of phosphate buffered solution (PBS) solution was added into each microtube and the mosquitoes were grinded using a pellet pestle. Ground mosquitoes were homogenized using buffer solution and the RNA were extracted according to the RNAeasy mini spin column (RNAeasy, Qiagen, Germany) manufacturer’s instructions. The microtubes containing extracted RNA were kept in -80°C freezer prior to PCR procedure.

The RNA extraction for the blood sample of the bats were conducted as follows: the FTA cards were first cut into 1 to 3 small pieces using paper puncher with diameter of 2 mm and were put into microplate, then 100 μl RNA rapid extraction solution (MagMAX^™^, Thermo Fisher Scientific, USA) were added into the microplate until the blood dissolved. The RNA extraction procedure for bat samples was performed according to manufacturer’s instructions for MagMAX Viral RNA Isolation Kit (Thermo Fisher, USA), using the dissolved blood samples from the FTA cards. The extracted RNA was kept in -80°C freezer prior to PCR procedure.

Reverse transcriptase polymerase chain reaction (RT-PCR) was used to detect the presence of JE virus using the following JE-specific primers: 5’-AGA GCG GGG AAA AAG GTC AT-3’ (forward) and 5’-TTT CAC GCT CTT TCT ACA GT-3’ (reverse) [35, 36]. The PCR reaction was performed as described previously in another study [37]. The RNA isolation procedure was done according to manufacturer’s instructions of the RT-PCR kit (Invitrogen, USA). Denaturation was done at 94°C for 15 seconds, continued with annealing at 55°C for 30 seconds, and extension at 68°C for 1 minutes. The procedure was repeated for 40 cycles. Final extension was done at 72°C for 5 minutes followed by holding at 12°C. The occurrence of a band at 162 bp in the electrophoresis (SYBR Safe, Invitrogen, USA) of PCR products was considered positive.

### Data analysis

To generate maps and to identify the distribution pattern of JE vectors and reservoirs using the average nearest neighbor (ANN) analysis [38], the software ArcGIS ver. 9.2 (Esri, New York) was used. The distribution pattern of the collected samples was categorized based on the nearest neighbor ratio (R) resulted in the ANN analysis as follows: clustered (R <1), random (R = 1), dispersed (R >1) [39]. We also performed the buffer analysis to visualize estimated flight ranges among captured mosquitoes and bats, resulting in as follows: *Culex* flight range of 2 km [40], Megachiropteran bats hunting range of 30 km, and Microchiropteran bats hunting range of 10 km [41, 42]. Minimum infection rate (MIR) of JE virus in mosquitoes was calculated as the ratio of the number of positive pools to the total number of mosquitoes tested using the PooledInfRate software (Biggerstaff, CDC, www.cdc.gov/ncidod/dvbid/westnile/software.htm). Statistical analysis used SPSS ver. 18.0 (SPSS, IL, USA). Bivariate analysis was performed to identify association between independent variables with JE virus infection using Chi-square or Fischer test, whenever appropriate. ANOVA test was performed to find mean differences of climate factors among three ecosystems. Multivariate logistic regression analysis was done with the forward stepwise method to identify independent associated risk factors of JE virus transmission in bats. Variables found to be statistically significant in bivariate analysis, and those that did not show statistical association yet were mentioned in other studies as risk factors of JE virus transmission, were put into the regression model. All variables demonstrating *p*-value of <0.05 (two-sided) were considered as statistically significant.

### Ethics statement

The ethical approval of current study was granted by the Medical and Health Research Ethics Committee (MHREC) of the Faculty of Medicine, Public Health and Nursing, Universitas Gadjah Mada (Ref. No. KE/FK/0339/EC/2020). Permission to publish the data was approved by the National Institute of Health Research and Development, Ministry of Health of Indonesia (Ref. No. 29011904–148). Research ethics related to the capture, specimen collection, maintenance, and release of bat samples were in accordance with the Animal Welfare Act 2006 [43].

## Results

**Fig 1:**
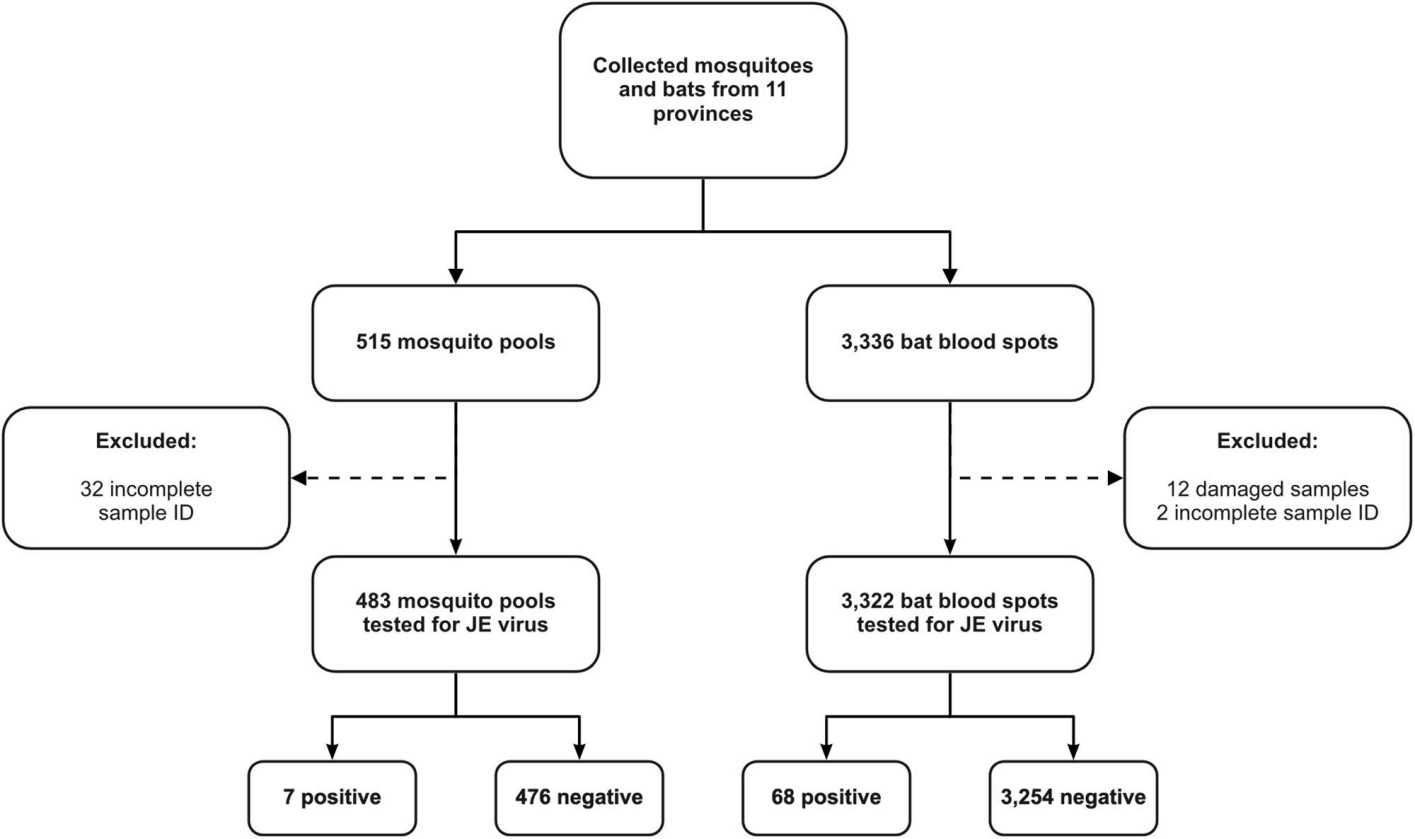
Study flow of the detection of JE virus in mosquito and bat samples in 11 provinces in Indonesia.

**Fig 2:**
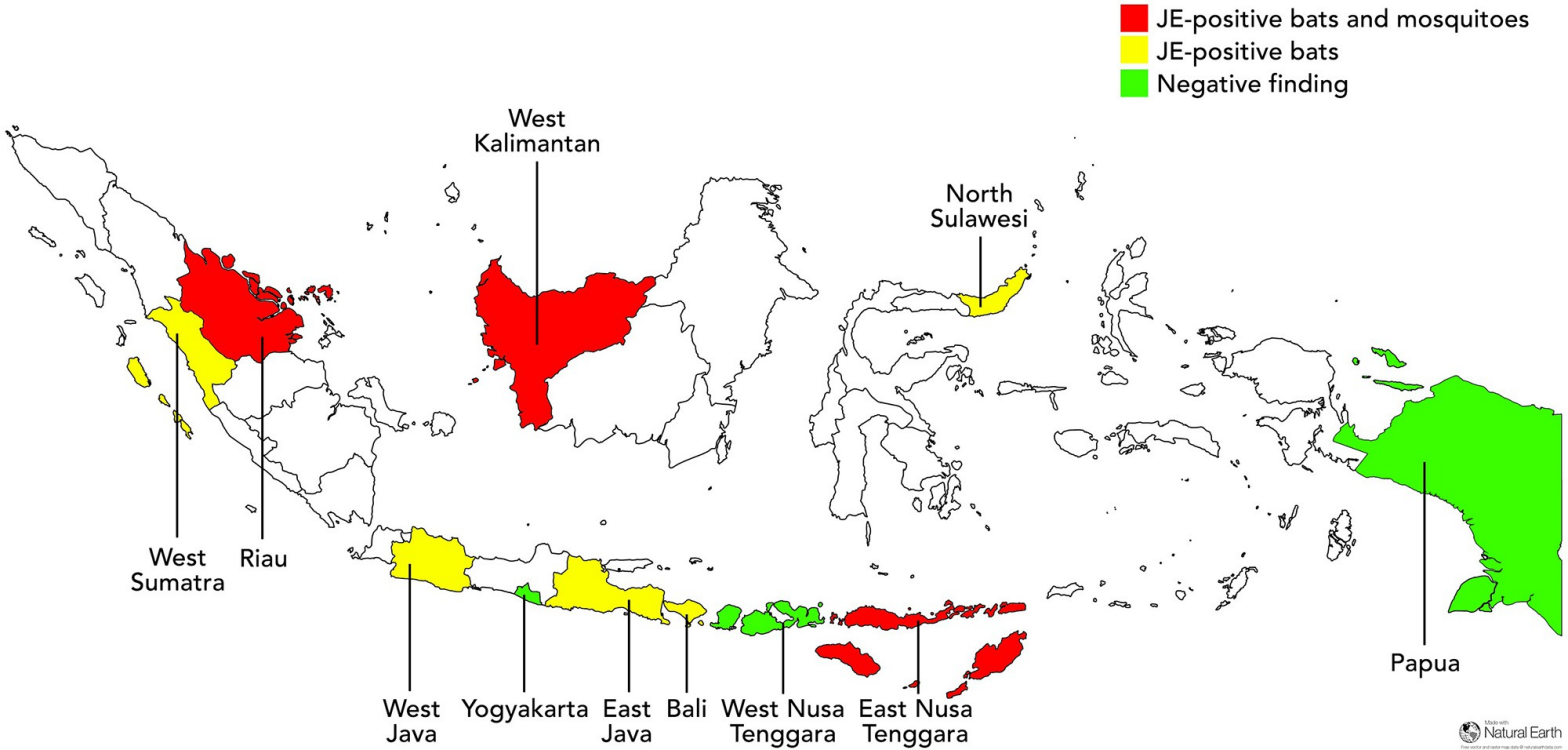
A map showing risk differences of JE virus transmission according to the provinces where human cases were previously detected by the Indonesian MoH and the results of JE virus detection in mosquitoes and bats in 11 provinces in Indonesia. Provinces with JE-positive bats and mosquitoes are shown in red shades, while provinces with only JE-positive bats are shown in yellow shades, and those with negative findings of JE virus are shown in green shades. Baseline map was made with Natural Earth (naturalearthdata.com).

Sample collection from 11 provinces resulted in a total of 483 mosquito pools and 3,322 blood spots of bats (Fig 1). Among 483 tested mosquito pools, 7 pools (1.4%) were positive for JE virus, and 68 out of 3,322 (2.0%) bat blood samples were found to be JE-positive (Table 1). In the current study, mosquitoes tested positive for JE virus belonged to the *Cx*. *tritaeniorhynchus* and *Cx*. *vishnui*. Mosquito samples found positive for JE virus were collected from the district of Bengkalis in Riau Province (1/21; 4.8%), district of Sambas in West Kalimantan Province (2/15; 13.3%), district of Kayong Utara in West Kalimantan Province (1/20, 5.0%), and the district of Ende in East Nusa Tenggara Province (3/13; 23.1%). Our results demonstrated MIR of *Cx*. *tritaeniorhynchus* of 2.16 in 1,000 mosquitoes, and MIR of *Cx*. *vishnui* of 0.22 in 1,000 mosquitoes. The JE-positive bats were collected from 8 provinces, namely West Sumatra (2/283; 0.7%), Riau (4/295; 1.4%), West Java (7/334; 2.1%), East Java (20/302; 6.6%), West Kalimantan (21/373; 5.6%), Bali (1/356; 0.3%), North Sulawesi (8/141; 5.7%), and East Nusa Tenggara (5/383; 1.3%). Study sites with both mosquitoes and bats found positive for JE virus are Riau, West Kalimantan, and East Nusa Tenggara (Fig 2).

**Table 1:** Total number of tested mosquito pools and bat blood samples according to provinces and ecosystems of collection.

Provinces and districts	Mosquitoes		Bats	
	Tested pools*	JE-positive^‡^	Tested samples	JE-positive^‡^
*West Sumatra*				
Forest	24 (10–25)	0	141	1 (0.7)
Coastal	18 (5–25)	0	64	0
Urban	12 (1–20)	0	78	1 (1.2)
*Riau*				
Forest	18 (5–25)	0	108	1 (0.9)
Coastal	15 (2–25)	0	81	1 (1.2)
Urban	22 (6–25)	1 (4.5)	106	2 (1.8)
*West Java*				
Forest	16 (3–25)	0	145	2 (1.3)
Coastal	9 (2–25)	0	131	5 (3.8)
Urban	7 (1–25)	0	58	0
*Yogyakarta*				
Forest	19 (5–25)	0	146	0
Coastal	19 (1–25)	0	92	0
Urban	12 (2–20)	0	123	0
*East Java*				
Forest	15 (1–25)	0	131	4 (3.0)
Coastal	7 (3–23)	0	106	3 (2.8)
Urban	11 (1–25)	0	65	13 (20.0)
*West Kalimantan*				
Forest	24 (10–25)	0	138	10 (7.2)
Coastal	15 (5–25)	2 (13.3)	138	5 (3.6)
Urban	14 (5–25)	1 (7.1)	97	6 (6.2)
*Bali*				
Forest	10 (4–25)	0	90	0
Coastal	12 (1–20)	0	159	1 (0.6)
Urban	8 (10–20)	0	107	0
*North Sulawesi*				
Forest	33 (15–25)	0	98	5 (5.1)
Coastal	10 (5–25)	0	17	2 (11.8)
Urban	13 (10–25)	0	26	1 (3.8)
*West Nusa Tenggara*				
Forest	11 (1–25)	0	97	0
Coastal	9 (1–25)	0	106	0
Urban	8 (6–25)	0	108	0
*East Nusa Tenggara*				
Forest	23 (1–25)	1 (4.3)	157	1 (0.6)
Coastal	8 (10–25)	2 (25.0)	151	1 (0.6)
Urban	5 (1–20)	0	75	3 (4.0)
*Papua*				
Forest	25 (1–25)	0	42	0
Coastal	14 (1–25)	0	78	0
Urban	17 (1–25)	0	63	0
**Total**	**483**	**7 (1.4)**	**3,322**	**68 (2.0)**

*number of tested mosquito pools (minimum to maximum number of mosquitoes tested in each pool)

^**‡**^shown in frequency (%)

The dominant ecosystem for mosquito collection was forest area (45.1%; *P =* 0.099). The majority of mosquito samples (90.3%) were collected in suburban areas. The collection sites include primary and secondary forests (28.4%) and farms (24.6%). Approximately 47.0% and 37.1% of mosquito samples were collected using outdoor human landing collection and animal-baited traps, respectively. Of these methods, 2 pools (28.6%) from outdoor collection and 5 pools (71.4%) from animal-baited trap collection were found positive for JE virus. All of the mosquito samples collected using indoor human landing method were found to be JE-negative. Statistical tests did not show any association among ecosystems, collection sites, and collection methods with JE virus transmission in the current study. The mosquito species positive for JE virus belonged to *Cx*. *tritaeniorhynchus* and *Cx*. *vishnui*. In the province of Riau, 1 pool of JE-positive *Cx*. *vishnui* was collected in a palm oil plantation near urban areas, while in West Kalimantan, 2 pools of JE-positive *Cx*. *vishnui* were collected in agricultural areas and in a coconut plantation near urban areas. Additionally, in West Kalimantan Province, 1 pool of JE-positive *Cx*. *tritaeniorhynchus* was collected in coastal area near human dwellings. All of the JE-positive samples (3 pools) collected in East Nusa Tenggara Province belonged to *Cx*. *tritaeniorhynchus* and were caught in lagoon and secondary forests. The total number of mosquitoes collected in the study was 27,648 mosquitoes, with *Cx*. *vishnui* being the most common mosquito species captured in all 3 ecosystems (S1 Table).

A total of 68 out of 3,322 blood samples (2.0%) were positive for JE virus in the current study, as shown in Table 2. Approximately 84.1% of total bat samples belonged to the adult group, with female to male ratio of 1:1.2. The JE-positive bats had lower median weight (32 g) and smaller bodies than that of JE-negative bats. The dominant ecosystem of bat collection was coastal area (18.5%), with lawns (31.1%) and farms (30.6%) located in close proximity with human dwellings being the most common collection sites. Statistical analysis did not show association between collection sites and JE virus transmission. Roughly 84.4% of the total collected bat sample genera belonged to the suborder Megachiroptera and Pteropodidae family: *Cynopterus*, *Dobsonia*, *Eonycteris*, *Macroglossus*, *Pteropus*, *Rousettus*, and *Thoopterus*. However, suborder and families of collected bats did not show statistical association with JE virus transmission in the current study. Among 68 JE-positive bats, 21 (30.9%) bats were collected in West Kalimantan Province and 20 (29.4%) bats were captured from East Java Province. All of the bat samples collected in Yogyakarta, West Nusa Tenggara and Papua Provinces showed negative results for JE virus. The majority of JE-positive bats belonged to the genus *Cynopterus* (44.1%) and *Macroglossus* (19.1%) (S2 Table).

**Table 2:** Basic characteristics of collected bats in study areas.

Parameter	Total^‡^	JE-negative^‡^	JE-positive^‡^	*P-*value
	N = 3,322	N = 3,524	N = 68	
Estimated age				
Juvenile	528 (15.9)	513 (15.8)	15 (22.1)	0.160**
Adult	2,794 (84.1)	2,741 (84.2)	53 (77.9)	
Sex				
Male	1,818 (54.7)	1,785 (54.9)	33 (48.5)	0.300**
Female	1,504 (45.3)	1,469 (45.1)	35 (51.5)	
Weight				
Mean (g)^†^	33 (1–780)	33 (1–780)	32 (3–92)	0.437^a^
≥50 g	851 (25.6)	837 (23.7)	14 (20.6)	0.337**
<50 g	2471 (74.4)	2417 (76.3)	54 (79.4)	
Ecosystems captured				
Forest	1293 (16.3)	1269 (16.2)	24 (20.6)	0.535**
Coast	1123 (18.5)	1105 (18.4)	18 (20.6)	0.196**
Urban	906 (16.5)	880 (16.7)	26 (7.4)	0.040**
Suborder				
Megachiroptera	2,805 (84.4)	2,744 (84.3)	61 (89.7)	0.224**
Microchiroptera	517 (15.6)	510 (15.7)	7 (10.3)	
Family				
Pteropodidae	2,805 (84.4)	2,744 (84.3)	61 (89.7)	0.226**
Vespertilionidae	290 (8.7)	285 (8.8)	5 (7.4)	0.684**
Hipposideridae	62 (1.9)	60 (1.8)	2 (2.9)	0.508*
Rhinolophidae	62 (1.9)	62 (1.9)	0	N/A
Emballonuridae	43 (1.3)	43 (1.3)	0	N/A
Molossidae	31 (0.9)	31 (1.0)	0	N/A
Nycteridae	28 (0.8)	28 (0.9)	0	N/A
Megadermatidae	1 (0.1)	1 (0.1)	0	N/A

^‡^presented in frequency (%)

^†^presented in median (min-max)

*Fisher’s exact test

**Chi square test

^a^Mann-Whitney test

Environmental factors including ambient temperature, relative humidity, and wind velocity were recorded hourly during sample collection in each collection sites. Mean temperature during sample collection period was observed the highest in the coastal areas (29.3°C), with the highest humidity observed in the urban areas (87.7%). Wind velocity was also found highest in the coastal areas (3.6 m/s). The ANOVA test showed mean differences in temperature, humidity, and wind velocity among all three ecosystems (*P* <0,001), as shown in Table 3. The results of the current study showed higher range of mean temperature in JE-positive mosquito collection sites (25.7–28.2°C) compared to that of JE-negative mosquito (22.2–24.3°C). JE-positive bats were captured in mean temperature of 27.5°C and mean relative humidity of 78.4%.

**Table 3:** Recorded environmental factors at sample collection sites.

Parameters^†^	Collection sites		
	Forest	Coastal area	Urban area
Temperature (°C)			
Minimum^§^	24.5±2.6	26.4±1.6	25.3±2.6
Maximum^§^	28.9±2.2	29.3±2.0	28.5±2.3
Humidity (%)			
Minimum^§^	76.9±11.2	75.6±9.4	78.1±12.1
Maximum^§^	87.1±9.0	85.3±9.3	87.7±7.9
Wind velocity (m/s)			
Maximum^§^	2.0±0.9	3.6±1.7	2.1±1.5

^†^presented in mean±SD

^§^observed mean differences; *p* <0.001 (ANOVA)

Multivariate analysis using the recorded variables of mosquito samples was unable to be performed since no statistically significant variables was found in bivariate analysis. Hence, multivariate analysis was done only to determine the transmission risk of JE virus in bats. Based on the results of the bivariate analysis and the literature review, we included the following variables into the regression model (Table 4): adult bats, capture location nearby urban areas, family of Pteropodidae, low bodyweight, low temperature, and high humidity at the collection site. Multivariate analysis demonstrated the following variables as independent risk factors of JE virus transmission in this study: collection sites near urban areas (AOR 2.02; 95% CI: 1.19–3.42) and high humidity (AOR 2.40; 95% CI 1.45–3.96).

**Table 4:** Independent risk factors of JE virus transmission in bats.

Variables	Univariate analysis		Multivariate analysis	
	*P-*value	COR (95% CI)	*P-*value	AOR (95% CI)
Adult bats	0.160**	0.66 (0.37–1.28)	-	-
Captured nearby human dwellings	0.027**	1.76 (1.06–2.92)	0.009	2.02 (1.19–3.42)
Pteropodidae family	0.226**	1.62 (0.73–3.56)	-	-
Low bodyweight (<50 g)	0.337**	1.33 (0.74–2.41)	-	-
Low temperature (<24°C)	0.186**	1.57 (0.80–2.96)	-	-
High humidity (>80%)	0.001**	2.31 (1.40–3.80)	0.001	2.40 (1.45–3.96)

COR: Crude Odds Ratio; AOR: Adjusted Odds Ratio; CI: confidence interval

*Fisher’s exact test

**Chi square test

**Fig 3:**
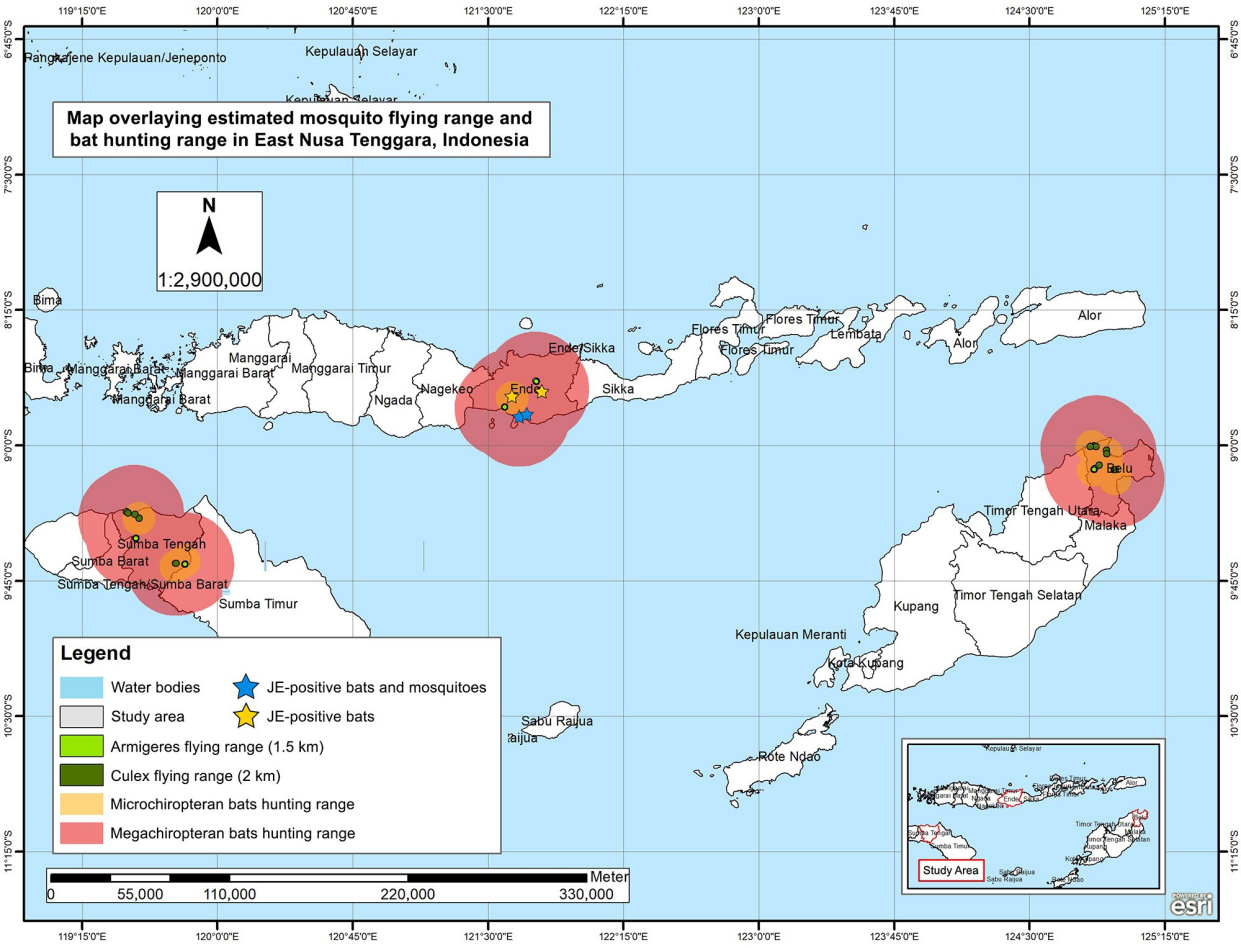
Map overlaying estimated mosquito flying range and bat hunting range in East Nusa Tenggara Province in Indonesia. Note the clustered distribution of JE-positive mosquitoes and bats in the same district of Ende. Map image was generated using ArcGIS (Esri).

We provided the sample map visualization of findings in East Nusa Tenggara Province, where all the JE-positive mosquitoes and bats were collected in the same district of Ende (Fig 3). Mapping results showed overlapping flight ranges among mosquitoes and bats from different collection sites, demonstrating the possibility of transmission and distribution of JE-positive samples in West Kalimantan, North Sulawesi, and Riau. The ANN analysis showed JE-positive mosquitoes and bats in dispersed pattern (R >1; *P* <0.001) in West Sumatra, Riau, North Sulawesi, and East Nusa Tenggara, whereas clustered pattern (R <1; *P* <0.05) was observed in East Java and West Kalimantan, and random distribution pattern (R = 1; *P* >0.05) was acquired in West Java.

## Discussion

The current study highlights the novel potential role of bats in the transmission of JE virus in Indonesia, shown by the presence of JE virus in bats and mosquitoes collected at the same site. The infection rate of mosquitoes reflects the prevalence of arboviral infection in mosquitoes, and the higher the infection rate, the higher the risk of human disease transmission in that respective area at that certain period of time [44]. This theory is, however, mostly valid at the time of mosquito collection and virus detection and may not always relfect the general transmission risk in that area. In the current study, mosquitoes tested positive for JE virus belonged to the *Cx*. *tritaeniorhynchus* and *Cx*. *vishnui*. These mosquito species have been identified as the principal vector of JE virus in Indonesia [45, 46], and have been collected in all of study sites in the current study. Additionally, higher MIR of *Cx*. *tritaeniorhynchus* than that of *Cx*. *vishnui* in this study demonstrated that the former mosquito species was the principal vector of JE virus. The study of MIR of JE virus in *Culex* mosquitoes has been limited. One study in India reported MIR of JE virus in *Cx*. *tritaeniorhynchus* of 0.97 and in *Cx*. *gelidus* of 0.68 in 1,000 mosquitoes [47]. Despite negative PCR findings in many mosquito pools, the presence of JE virus in the mosquito population should not be ruled out considering the sampling technique of entomological survey that might not facilitate the collection of all mosquito populations in the surveyed sites [48].

Since the results of the current study showed the majority of JE-positive bats derive from the Megachiroptera suborder, this might have demonstrated one of the first molecular evidence of JE virus infection in Megachiropteran bats. Previous study findings in Indonesia supported such results, showing that antibodies against JE virus were found in *Pteropus* spp., with seroprevalence of 4.2% (3/70) [49]. The detection of antibodies against JE virus in bats has been reported in other studies, particularly in Microchiropteran bats from the genera *Hipposideros*, *Myotis*, *Miniopterus*, *Rhinolophus*, and *Vespertillio* [12, 50–53]. The molecular detection of JE virus in bats has also been studied in Microchiropteran bats in China, demonstrating that *Myotis ricketti*, *M*. *schreibersii*, *Murina aurata*, *R*. *leschenaultia*, and *R*. *affinis* were found positive for JE virus [54, 55]. The presence of antibodies in bats represents past infection of the virus and indicates that active infection of the virus remains questionable. Hence, the detection of the virus itself in bats better characterizes active infection and thus the potential role of bats in the transmission of arboviruses [56]. Additionally, JE virus in bats may undergo viral overwintering that can cause the virus to exist inside the bats for over 15 weeks in laboratory settings [12, 57], while in normal condition the high viremia was found to last as long as 30 days that the bats are considered the most infectious during this period [57].

Uninfected mosquitoes can potentially be infected by the circulating JE virus in bats’ circulation during blood feeding episodes [58]. There are several theories in regard to JE virus transmission from mosquitoes to bats, and vice versa: 1) insectivorous bats get infected through ingestion of JE-infected mosquitoes [59]; 2) frugivorous bats get infected through JE-infected mosquito bites [58]; and 3) mosquitoes get infected from sucking half-eaten fruit contaminated with saliva from JE-infected bats [60]. As in the current study, the majority of JE-positive bats belonged to the frugivorous Megachiroptera suborder, hence the likeliest mechanisms of JE virus transmission were that the bats got infected through JE-infected mosquitoes. Such mechanism was evidenced by another study in Thailand where all blood meals in JE-infected *Culex* mosquitoes were blood from the fruit bat *Rousettus leschenaultii* [58]. Another laboratory experiment showed that in *Cx*. *annulirostris* inoculated with JE virus, the mosquitoes were able to transmit the virus into *Pteropus alecto* and were able to induce viremia [15]. These results strengthen the plausible mechanisms of JE virus maintenance in bats, and their potential role as reservoir hosts of JE virus [61]. Unfortunately, sequencing analysis of the blood meal and host preference in mosquitoes was not performed in the current study, making determination of plausible infection mechanism difficult.

Mosquito-borne diseases are vulnerable to environmental changes, mostly due to the altered survival ability of mosquitoes that is generally affected by ambient temperature and humidity [62]. The results of the current study showed higher range of mean temperature in JE-positive mosquito collection sites compared to that of JE-negative mosquito. The impacts of high temperature in mosquito survival are as follows: 1) shortened development time from eggs to adults, hence smaller body and may affect the ability in harboring arboviruses due to immaturity of midgut and salivary barriers [63]; and 2) shortened gonotrophic cycle, hence increasing the frequency of blood feeding and mosquito-host contact [64]. High temperature and low humidity cause increased metabolic rate of mosquitoes, leading to increased evaporation and shorter mosquito survival [65], and reduced flight range [40]. Additionally, wind velocity of over 2 m/s aids flight range of mosquito, so that they could have been blown by the wind into more dispersed areas to transmit disease [66], or blown into the bats’ habitat.

Shifts in land use and increased urbanization can play a role in the expansion of mosquito and bat distribution closer to urban areas where JE transmission can occur [67–69]. Habitat disruption due to human actions can alter mosquito oviposition sites, leading to shifts in species abundance, and later, behavioral pattern changes [20, 64]. According to the land use maps, the results of the current study demonstrated capture of JE-positive bats in urban areas in West Sumatra, East Java, West Kalimantan, Bali, North Sulawesi, and East Nusa Tenggara. Similarly, the majority (79.4%) of the JE-infected bats from genera *Cynopterus*, *Eonycteris* and *Macroglossus* were collected in plantations and urban areas. These bat genera are commonly found in tropical forests and caves, and are not generally found in close proximity to human dwellings [70]. Habitat change also affects bats’ behavior, including finding prey during daytime when humans’ activities peak [71, 72], leading to intensive bat-human contact and increased risk of viral spillover. In this study, JE-positive mosquitoes were collected from the same environment with JE-positive bats in Riau, West Kalimantan and East Nusa Tenggara, showing plausible mechanisms of high-risk viral transmission between mosquitoes and bats in these provinces. Multivariate analysis also demonstrated that collection sites nearby human dwellings was an independent risk factor of JE virus transmission in this study. This should increase awareness of potential impact of zoonotic pathogens on human health in the community [73, 74].

The current study visualized the distribution of JE-infected mosquitoes and bats according to their distribution using ANN analysis. The ANN analysis can assist to determine specific areas of high disease transmission risk, particularly in clustered patterns where focused disease control and prevention measures are encouraged for better resource allocation [75, 76]. Such results will also affect the strategies implemented by local governments in JE control and prevention. Other studies have also showed the use of ANN analysis in dengue control [39, 77], demonstrating that clustered distribution pattern was more convenient to implement disease control program when compared to dispersed and random patterns. In this study, disease control should theoretically be uncomplicated in the provinces of West Sumatra, Riau, North Sulawesi, and East Nusa Tenggara, where clustered patterns of JE virus in bats and mosquitoes were observed. Furthermore, buffer mapping also aids in visualizing the flight ranges of mosquitoes, in order to estimate potential locations for oviposition and traps, as well as potential areas for environmental modifications [78, 79], and are more commonly used in surveillance of malaria and dengue. In terms of bats, buffer mapping is useful for picturing their flight ranges, so that the roosting habitats can be estimated [80, 81]. The results of the current study showed overlapping of bat and mosquito flight ranges among JE-infected sample collection sites, demonstrating the possibility of viral spillover to other areas, including human dwellings. Similar findings have also been found in West Kalimantan province in our previous pilot study [25].

The current study has brought into light the potential involvement of bats in the local transmission of JE virus in Indonesia. Bats may serve as either maintenance host or reservoir host, depending on the presence of pigs in that respective area [61, 82]. The presence of JE-positive vectors and reservoirs should alarm the local governments to formulate JE control and prevention strategies. Since environmental factors play a major role in the transmission of JE virus too, the One Health concept should be encouraged to aid in multidisciplinary approaches to combat JE [83, 84]. Local risk stratification scores can also be established to estimate high risk areas for JE virus transmission, hence suitable disease control and prevention programs can be put into action according to the risk. While the WHO recommended JE vaccination as the most important intervention to control JE [85], other complementary strategies may include strategic efforts in vector and reservoir control, habitat conservation, restriction of land use, and capacity building in disease surveillance [86]. In the absence of routine JE vaccination program in many provinces in Indonesia, the current study findings can aid in the coverage expansion of the ongoing JE sentinel surveillance by the Ministry of Health of Republic of Indonesia, in line with the improvement of JE vaccination campaign and the intensification of active case finding and entomological surveys in provinces with JE-infected mosquitoes and bats. Medical and health professionals working in endemic areas of JE should also be aware of the risk of JE virus transmission; therefore, it is recommended to use better judgment in the diagnosis of patients presenting with acute encephalitis syndrome. At the most, the detection of JE virus RNA at 8 out of 11 study provinces in Indonesia in the current study underlines the likely ongoing risk of JE virus, and its potential implications on human health.

The current study has several limitations. First, the nature of the surveillance study in very short period of time conducted in selected areas might have affected the general results of JE-virus positivity. Second, sequencing study was not performed in the current study. However, results from this study should benefit the stakeholders in areas with similar situation or potential transmission risk, to adjust the JE prevention and control program according to local condition.

## Supporting information

S1 Fig Mapping of overlapping flight ranges among mosquitoes and bats, demonstrating the possibility of transmission of JE virus in the province of Riau. Map image was generated using ArcGIS (Esri).
(DOCX)

S1 Table Total number of collected mosquito species according to collection ecosystems. (DOCX)

S2 Table Collected JE-positive bat species according to collection ecosystems. (DOCX)
