# Supplementary material for: Estimation of Japanese encephalitis virus infection prevalence in mosquitoes and bats through nationwide sentinel surveillance in Indonesia
Source: PLoS One. 2022 Oct 12;17(10):e0275647. doi: 10.1371/journal.pone.0275647 (PMC9555671; doi:10.1371/journal.pone.0275647)

**S1 Fig.** **Mapping of overlapping flight ranges among mosquitoes and bats, demonstrating the possibility of transmission of JE virus in the province of Riau.** Map image was generated using ArcGIS (Esri).


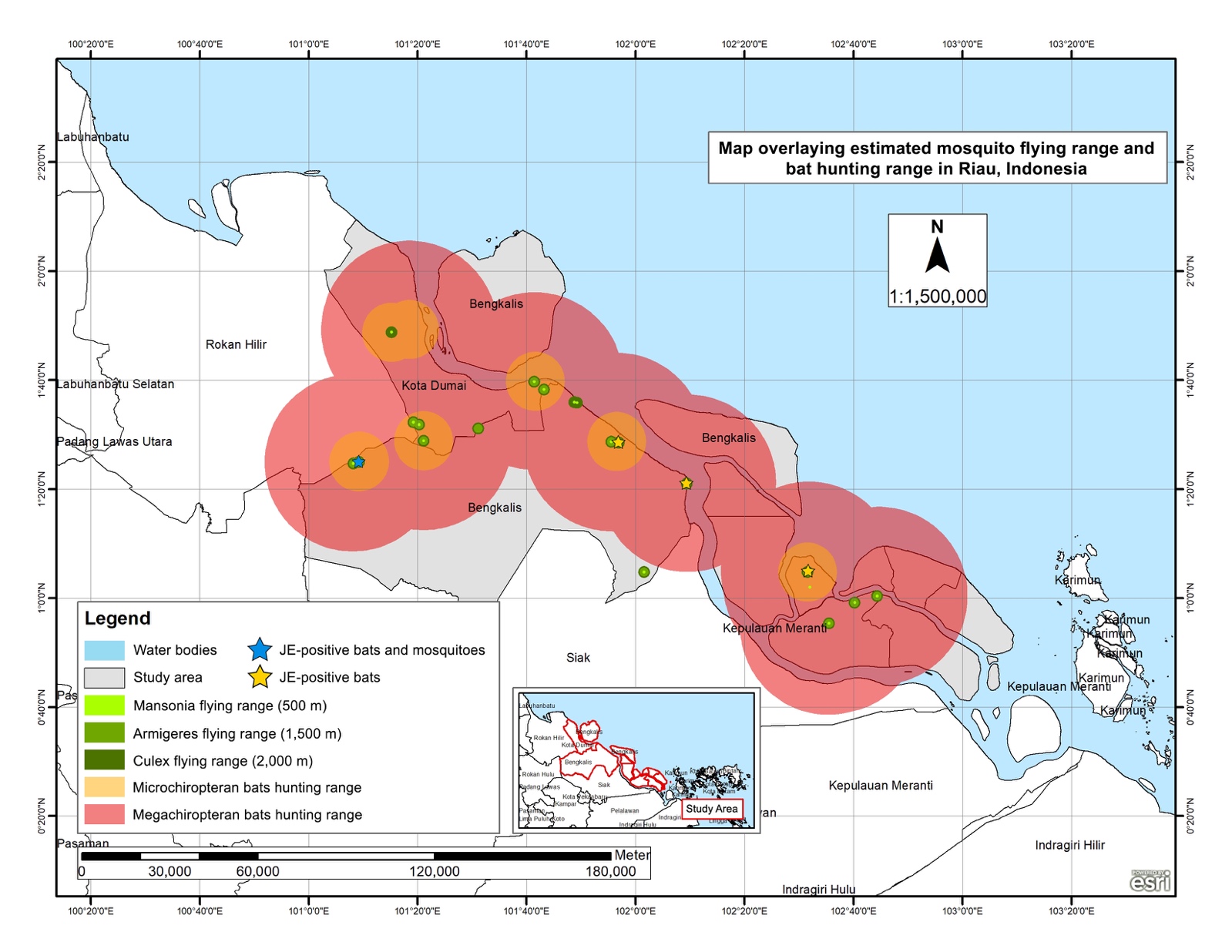

Supplement: S1 Fig — Map image was generated using ArcGIS (Esri). (DOCX) [file pone.0275647.s001.docx]
