# Supplementary material for: Estimation of Japanese encephalitis virus infection prevalence in mosquitoes and bats through nationwide sentinel surveillance in Indonesia
Source: PLoS One. 2022 Oct 12;17(10):e0275647. doi: 10.1371/journal.pone.0275647 (PMC9555671; doi:10.1371/journal.pone.0275647)
Supplement: S1 Table — (DOCX) [file pone.0275647.s002.docx]

**S1 Table. Total number of collected mosquito species according to collection ecosystems.**

| **No.** | **Mosquito species** | **Ecosystems of collection** | | | **Total** |
| --- | --- | --- | --- | --- | --- |
|  |  | **Forest** | **Coastal area** | **Urban area** |  |
| 1. | *Aedes albopictus* | 3 | 0 | 5 | 8 |
| 2. | *Ae. andamanensis* | 0 | 95 | 21 | 116 |
| 3. | *Ae. annandalei* | 5 | 0 | 0 | 5 |
| 4. | *Ae. poicilius* | 2 | 2 | 13 | 17 |
| 5. | *Ae. vexans* | 0 | 257 | 1 | 258 |
| 6. | *Anopheles bancroftii* | 176 | 2 | 1 | 179 |
| 7. | *An. farauti* | 15 | 71 | 43 | 129 |
| 8. | *Armigeres kesseli* | 282 | 24 | 282 | 588 |
| 9. | *Ar. malayi* | 78 | 0 | 0 | 78 |
| 10. | *Ar. subalbatus* | 491 | 151 | 275 | 917 |
| 11. | *Coquillettidia crassipes* | 47 | 5 | 1 | 53 |
| 12. | *Culex bitaeniorhynchus* | 35 | 54 | 1 | 90 |
| 13. | *Cx. fuscocephalus* | 6 | 20 | 1 | 27 |
| 14. | *Cx. gelidus* | 263 | 248 | 199 | 710 |
| 15. | *Cx. hutchinsoni* | 1 | 0 | 0 | 1 |
| 16. | *Cx. pseudosinensis* | 0 | 1 | 0 | 1 |
| 17. | *Cx. quinquefasciatus* | 419 | 680 | 529 | 1,628 |
| 18. | *Cx. sinensis* | 84 | 0 | 0 | 84 |
| 19. | *Cx. sitiens* | 1,017 | 437 | 42 | 1,496 |
| 20. | *Cx. tritaeniorhynchus* | 1,082 | 527 | 237 | 1,846 |
| 21. | *Cx. vishnui* | 6,920 | 4,472 | 2,478 | 13,870 |
| 22. | *Mansonia annulifera* | 813 | 0 | 119 | 932 |
| 23. | *Mn. bonneae* | 388 | 514 | 1 | 903 |
| 24. | *Mn. dives* | 253 | 22 | 0 | 275 |
| 25. | *Mn. indiana* | 0 | 0 | 4 | 4 |
| 26. | *Mn. uniformis* | 3,159 | 157 | 117 | 3,433 |
|  | **Total** | **15,539** | **7,739** | **4,370** | **27,648** |
