# Supplementary material for: Estimation of Japanese encephalitis virus infection prevalence in mosquitoes and bats through nationwide sentinel surveillance in Indonesia
Source: PLoS One. 2022 Oct 12;17(10):e0275647. doi: 10.1371/journal.pone.0275647 (PMC9555671; doi:10.1371/journal.pone.0275647)
Supplement: S2 Table — (DOCX) [file pone.0275647.s003.docx]

**Table S2. Collected JE-positive bat species according to collection ecosystems.**

| No. | Bat species | Ecosystems of collection | | | Total |
| --- | --- | --- | --- | --- | --- |
|  |  | Forest | Coastal area | Urban area |  |
| 1. | *Cynopterus brachyotis* | 6 | 6 | 7 | 19 |
| 2. | *C. minutus* | 0 | 1 | 0 | 1 |
| 3. | *C. nusatenggara* | 0 | 0 | 1 | 1 |
| 4. | *C. sphinx* | 2 | 2 | 3 | 7 |
| 5. | *C. titthaecheilus* | 1 | 0 | 1 | 2 |
| 6. | *Eonycteris maculata* | 3 | 0 | 0 | 3 |
| 7. | *E. spelaea* | 2 | 2 | 2 | 6 |
| 8. | *Hipposideros galeritus* | 0 | 0 | 1 | 1 |
| 9. | *H. madurae* | 1 | 0 | 0 | 1 |
| 10. | *Kerivoula hardwickii* | 1 | 0 | 2 | 3 |
| 11. | *Macroglossus minimus* | 3 | 1 | 4 | 8 |
| 12. | *M. sobrinus* | 1 | 2 | 2 | 5 |
| 13. | *Pipistrellus javanicus* | 0 | 0 | 1 | 1 |
| 14. | *Rousettus amplexicaudatus* | 0 | 3 | 0 | 3 |
| 15. | *R. celebensis* | 1 | 1 | 0 | 2 |
| 16. | *Scotophilus kuhlii* | 0 | 0 | 1 | 1 |
| 17. | *Thoopterus nigrescens* | 3 | 0 | 1 | 4 |
|  | Total | 24 | 18 | 26 | 68 |
